# Psychological distress by age at migration and duration of residence in Sweden

**DOI:** 10.1016/j.socscimed.2020.112869

**Published:** 2020-04

**Authors:** Helena Honkaniemi, Sol Pía Juárez, Srinivasa Vittal Katikireddi, Mikael Rostila

**Affiliations:** a Department of Public Health Sciences, Stockholm University, Stockholm, Sweden; b Centre for Health Equity Studies (CHESS), Stockholm University/Karolinska Institutet, Stockholm, Sweden; c MRC/CSO Social and Public Health Sciences Unit, University of Glasgow, Glasgow, UK

**Keywords:** Age at migration, Duration of residence, Integration, Life course theory, Migrant, Psychological distress, Sweden

## Abstract

Migrants suffer from worse psychological health than natives in many countries, yet the extent to which this varies by age at migration and duration of residence in the receiving context remains unexplored in Sweden. Drawing on a life course approach, we investigate differences in psychological distress by age at migration and duration of residence in working-age migrants to Sweden, and examine the role of various social determinants of health in explaining these differences relative to Swedish-born.

Using pooled cross-sectional data from the 2011/2015 *Health on Equal Terms* survey in Västra Götaland Region, Sweden (n = 58,428), we applied logistic regression analysis to calculate predicted probabilities and average marginal effects (AME) of migrant status, by age at migration and duration of residence, on psychological distress. Analyses were stratified by sex and region of origin and controlled for indicators of socioeconomic status (SES), social cohesion, and discrimination to assess their potential contribution to differences in migrants' and natives' psychological distress.

All migrants except men from OECD-predominant regions had a greater probability of psychological distress than Swedish-born (ranging from AME 0.031 [95% Confidence Interval or CI 0.000–0.062] for OECD women to AME 0.115 [95% CI 0.074–0.156] for non-OECD men). Marginal effects of migration status on psychological distress probabilities generally increased with age at migration and duration of residence. Differences between migrants and natives were largely attenuated after controlling for social determinants, the greatest contribution coming from inequalities in social cohesion, followed by inequalities in discrimination and SES.

Our results suggest a relative health advantage of early-life compared to later-life migration, albeit with worse outcomes with longer residence in Sweden. The predominance of integration opportunities in childhood strengthens calls for supportive policies to assist older migrants' integration directly upon arrival, which may ultimately improve their psychological wellbeing.

## Highlights

• International migrants often suffer from worse psychological health than natives.
• Psychological distress increases with age at migration and duration of residence.
• Native-migrant differences are largely accounted for by social inequalities.
• Inequality is found in social cohesion, discrimination, and socioeconomic status.

## 1 Introduction

The *healthy migrant paradox* stipulates that international migrants experience better physical health than natives (Abraido-Lanza et al., 1999; Aldridge et al., 2018; Kennedy et al., 2015), yet findings for psychological health largely suggest a migrant disadvantage (Close et al., 2016). In Sweden, evidence on first- and second-generation migrants have revealed increased risks of affective, neurotic, and psychotic disorders (Hollander et al., 2016; Westman et al., 2006a); substance use disorders (Manhica et al., 2016, 2017); suicide (Di Thiene et al., 2015; Westman et al., 2006b); and underlying symptoms of psychological distress (Kosidou et al., 2012; Sundquist et al., 2000; Wamala et al., 2007). In order to better understand these outcomes, migrant populations should be considered in relation to the timing of their migration, including their age at migration and duration of residence in the receiving country, as well as their experiences across sending, transit, and receiving contexts (Acevedo-Garcia et al., 2012). Together, these measures can help capture the health-modifying role of age- and duration-specific exposures to new contexts.

### 1.1 Psychological distress and the timing of migration

Psychological distress is a useful measure to capture affective symptomologies of varying severity, from the sub-clinical to cases manifesting as psychiatric disorders (Mirowsky and Ross, 2003; Payton, 2009). It is effective for assessing population-wide patterns of poor mental health (Drapeau et al., 2012), including the mental health of migrants. An international review on migrants' psychological distress found that age at migration and duration of residence in the receiving country were among the many migration-specific factors associated with distress (Jurado et al., 2017). The direction of these associations appears to vary by context. Evidence from North America has revealed greater odds of psychological distress, as well as mood and anxiety disorders, among childhood rather than adult migrants (Breslau et al., 2009; Gong et al., 2011; Islam et al., 2014; Patterson et al., 2013; Perreira et al., 2015; Yang, 2019), contradicting European studies that indicate increased odds of depressive symptoms among older arrivals (Lanari and Bussini, 2012; Lanari et al., 2018). Both the North American (Breslau et al., 2009; Perreira et al., 2015) and European literature (Johnson et al., 2017; Jurado et al., 2014; Lanari and Bussini, 2012) have found increases in poor mental health with longer duration of residence. While it appears that both age and duration can modify the health risks posed by migration status, their joint influence on migrant psychological health across the life course remains unexplored in Sweden.

### 1.2 Applying a life course approach

Life course theory is a multidisciplinary approach to examining how social conditions are embodied throughout the lifespan to culminate in health (Ben-Shlomo et al., 2014; Chandola et al., 2014; Kuh et al., 2003). Within migration research, it can be used to explore specific developmental windows during which the experience of migration and its accompanying stressors are vital for long-term health (Acevedo-Garcia et al., 2012). A number of alternative theoretical models have been put forth in the literature to explain the causal pathways between exposures, such as the migration experience, and health outcomes.

The *sensitive period model* suggests that certain social and environmental exposures have amplified health effects during specific life periods (Ben-Shlomo et al., 2014). Rather than being a proponent of causal determinism (i.e., that exposure during these periods is necessary for an outcome to arise), the model describes a susceptibility to exposures. Thus, if migration at certain ages were to increase the probability of future psychological distress via so-called effect modification (Kuh et al., 2003), independent of the duration or quality of residence in the receiving country, its effects would conform to the sensitive period model.

Instead of being fixed upon exposure, however, health effects are more likely to be a consequence of mediating and modifying factors arising throughout migration. According to the *accumulation of risk model*, sequences of social exposures such as education, employment, and social connection (Åslund et al., 2009) can additively influence health (Kuh et al., 2003). Alternatively, the *chain of risk model* suggests that initial exposures can trigger a “chain reaction” of events culminating in a single health effect (Ben-Shlomo et al., 2014). Unlike the sensitive period approach, these models highlight the importance of the period arising after initial exposure. Thus, arrival and integration at different points in a migrant's life may render them more or less susceptible to certain health risks not in and of themselves, but due to the duration and quality of subsequent residence in the receiving country. This would suggest that both age at migration and duration of residence, as well as the social determinants of health arising during integration, should be empirically assessed to explore the life course models at play.

### 1.3 Integration processes

Within the life course framework, integration processes may entail various types of “age-salient life challenges” (Yang, 2019). Migration during childhood presents unique opportunities for integration through educational immersion, cultural exposure, and language acquisition, in turn facilitating migrants' long-term health (Böhlmark, 2009; Hermansen, 2017; Åslund et al., 2009). Migrants arriving as adults may have more limited prospects for social connection and employment due to various cultural and linguistic barriers, relying instead on support from within ethnic communities (Edin et al., 2003; Rostila, 2010). While membership in such communities has been affiliated with lower socioeconomic status (SES) (Rostila, 2010), some evidence suggests a protective effect of the so-called social cohesion fostered within them (Edin et al., 2003; OECD, 2015). All of these individual factors should be considered within the scope of broader social determinants pertaining to culture (Berry et al., 1987; Detollenaere et al., 2018), geopolitics (Close et al., 2016), and migration policy (Juárez et al., 2019) across the various stages of migration (Acevedo-Garcia et al., 2012), to help identify sensitive periods in the life course during which migration can set off a trajectory of health-altering social conditions. Particular emphasis should be placed on the integration processes of working-age migrants, who comprise the bulk of foreign-born persons in Sweden (Statistics Sweden, 2019a) and whose socioeconomic stability is advantageous to foreign- and native-born alike (Portes, 2019).

### 1.4 Study aim

The overall aim of our study is to determine how symptoms of poor mental health, specifically psychological distress, differ among working-age migrants by their age at migration and duration of residence, relative to native Swedes. We will also investigate whether native-migrant health differences are accounted for through concurrently-measured social determinants of health. The purpose will be to explore, rather than confirm, the role of different life course models. Based on the prior evidence, we hypothesize that migrants experience more psychological distress than natives overall, with inequalities widening by older age at migration due to limited opportunities for socioeconomic and cultural integration, and increasing with longer duration of residence through the accumulation of integration-related risk.

## 2 Methods

### 2.1 Study context

Sweden has long been characterized by a generous migration policy. Migration in the mid-20^th^ Century primarily consisted of foreign labor from Finland and Southern Europe, but by the 1980s and 1990s various international conflicts led Sweden to accept over 100,000 asylum seekers from Iran, Iraq, and former Yugoslavia. Since then, the country has witnessed both increased labor migration within the European Union (EU) as well as new waves of asylum seekers from the Middle East (Swedish Migration Agency, 2019). Today, 19% of the Swedish population is foreign-born, half of whom are women. At the Swedish national and Västra Götaland (VG) county levels, similar shares originate from Organization for Economic Cooperation and Development (OECD; 9%) and non-OECD regions (10%) (Statistics Sweden, 2019b), reflecting the relative generosity of Swedish migration policy and its weak selection against asylum seekers and refugees.

### 2.2 Study sample

Data were drawn from the *Health on Equal Terms* (HET) survey collected by Statistics Sweden (Public Health Agency of Sweden, 2019). The HET is a population-representative cross-sectional survey conducted every four years, with additional data available through register linkage (Statistics Sweden, 2015a). Using a sampling frame designed with national register data, respondents are randomly selected for one national sample and multiple county-level supplements. Survey data were available for respondents residing in VG County in Southwestern Sweden in 2011 and 2015, with linked register data from two years prior to each survey year (i.e., the most recently published data at the time of linkage) (Statistics Sweden, 2015a, 2015b). 2011 survey data were based on the supplementary HET selection within VG that were sent the national form (n = 19,048) or a regional test form (n *=* 76,587; *differences in the two forms are not applicable to this study*), with a total response rate of 54.3% (n = 51,930; from 32.3% for non-European migrants to 58.2% for Nordic-born, including Swedish-born respondents) (Statistics Sweden, 2011a, 2011b). For 2015, respondents drawn from both the national (n = 20,000) and supplementary (n = 91,347) samples yielded a total response rate of 55.5% (n = 52,348) (Statistics Sweden, 2015a, 2015b), with response patterns by nativity reflecting those in 2011 and comparable Swedish surveys (Svensson et al., 2012). Information on reasons for non-response is unavailable. Both years included a panel of 11,817 respondents residing in VG county, of which only the most recent (2015) data were included in this study. We finally restricted the sample to those residing in VG county (16–84 years; n = 87,512) and of working age (16–64 years; n = 58,428; see Fig. S1 for participant selection).

### 2.3 Variables

#### 2.3.1 Outcome variable

Psychological distress was measured using the 12-item General Health Questionnaire (GHQ-12), commonly used to capture symptoms of non-psychotic mental disorders such as depression and anxiety in both general (Drapeau et al., 2012; Lundin et al., 2016; McDowell and Newell, 1996) and migrant populations (Brydsten et al., 2019; Gotsens et al., 2015; Sidorchuk et al., 2017). The GHQ-12 consists of items pertaining to various psychological symptoms experienced in the past two weeks, including one's ability to concentrate on daily activities, to sleep, to manage problems, and to make decisions, alongside feelings of accomplishment, satisfaction, and happiness, versus feelings of dejection or depression, tension, faithlessness, and worthlessness. Symptom frequency was ranked on a four-point Likert scale with categories collapsed and recoded dichotomously (“more than usual” and “as usual”, “less than usual” and “much less than usual”). All items were summed into a 12-point summary score with high internal validity (Cronbach's α = 0.909). Given a skewed distribution and the expectation of a non-linear association, the scale was dichotomized with a cut-off of more than three points indicating the presence of distress (Boström and Nyqvist, 2010; Brydsten et al., 2019).

#### 2.3.2 Primary explanatory variables

Respondents were distinguished by nativity, where Swedish-born comprised the reference group. Due to lack of more explicit country-of-birth categories or reasons for migration, we divided those born outside Sweden into two sub-groups based on their region of origin (Rostila, 2007). *Migrants from OECD regions* consisted of respondents from regions predominantly composed of OECD countries, i.e., the Nordic region (outside Sweden), Europe (outside the Nordics), North America, and Oceania. The assumption was that these migrants were less likely to experience adverse social conditions both before and after migration compared to their non-OECD counterparts, with cultural backgrounds similar to those of Swedish-born. *Migrants from non-OECD regions* included respondents from regions not predominantly composed of OECD countries, i.e., Africa, Asia, South America, and regions marked as 'Other'. We assumed that respondents in this population were more likely to have experienced economic or humanitarian hardship, with more culturally distinct backgrounds relative to Swedish-born.

**Age at migration** corresponded to the age at which a migrant was registered as a resident in Sweden. As the primary focus of the study was on age at migration, we coded age categories in intervals of *0-12, 13–17, 18–24, 25–34,* and *35–64 years* based on relevant life stages pertaining to childhood, adolescence, enrolment in higher education, labor market attachment, and family formation, respectively (Rumbaut, 2004); as well as previous evidence of health differences by age at migration (Breslau et al., 2009; Gong et al., 2011; Leão et al., 2009; Leu et al., 2008; Li and Wen, 2015; Patterson et al., 2013). **Duration of residence** at the time of the survey was dichotomized into *less than 15 years* and *more than or equal to 15 years*, based on previous operationalizations (Juárez et al., 2018) and to ensure statistical power. A combined variable reflecting both age at migration and duration of residence was also created to avoid issues of multicollinearity.

#### 2.3.3 Mediators

We additionally included common indicators of migrant integration (Ager and Strang, 2008), conceptualized here as social determinants of health among both natives and migrants (Castañeda et al., 2015), to incrementally examine their potential role in mediating psychological differences by the timing of migration.

**Socioeconomic status:** *Educational level* or highest achieved education was classified as low (i.e., up to two years of upper secondary education), medium (i.e., up to two years of university college), and high (i.e., graduate and postgraduate studies). Given a large portion of missing data for employment status, we examined salary- and social benefit-based *individual* and *household disposable income* to proxy individual employment status and availability of financial resources, respectively, dividing each variable into quintiles for comparative purposes (Brydsten et al., 2019). Finally, we included dichotomous measures of *civil status* (single or married/cohabiting) and *Swedish citizenship status* (Swedish citizen or not; *not equivalent to lawful residence*). All of the above were acquired from Swedish population registers, and include a ‘missing’ category to capture recent migrant arrivals, who were less likely to have a complete register profile.

**Social cohesion:** We additionally examined the qualitative components of social relationships (rather than quantitative, i.e., network size) as operationalized in previous research (Brydsten et al., 2019). We included two dichotomized indicators of individual social cohesion, *instrumental (practical)* *support* and *emotional (social)* *support*. In addition, we captured contextual social cohesion using a dichotomized indicator of *general trust* and a summary measure of *social participation* indicating whether the respondent reported having participated in at least one of 15 social activities (e.g., a study group, religious gathering, family reunion, etc.) in the past 12 months (Cronbach's α = 0.686).

**Discrimination:** We designed a dichotomous measure to capture experiences of *perceived discrimination* in the past three months, focusing on incidents targeting the respondent's “ethnic affiliation”, “religion”, or “skin color” (Cronbach's α = 0.518). Indirect measures of discrimination included *fear of going out alone*, *exposure to threats*, and *exposure to physical violence*, each of which was also dichotomized. All measures of discrimination and social cohesion were drawn from the survey (see Table S1 for survey items).

### 2.4 Statistical analysis

The study analyzed pooled cross-sectional data from one-time HET respondents in 2011 and 2015, as well as 2015 data from HET panel respondents, stratifying by sex and nativity. Crude logit models were calculated to compare psychological distress in migrants and Swedish-born, by age at migration and duration of residence separately, then together. We controlled for various covariates to capture changes in the models' goodness-of-fit to psychological distress. All stages of analysis were additionally controlled for age at the time of the survey and panel membership, the latter to facilitate inclusion of panel data and account for any unobserved differences between one-time and panel respondents.

Statistical analysis was conducted using Stata/MP 14.2. Survey weights were applied using the *pweight* command to account for stratified sampling and sociodemographic differences in respondents and non-respondents. To facilitate comparison across logit models (Mood, 2010), we applied the *margins* command to calculate predicted probabilities and average marginal effects (AME; i.e., the average effect of the exposure on the probability of the outcome) using marginal standardization, an endorsed method for making population-wide inferences (Muller and MacLehose, 2014). Corresponding 95% confidence intervals (CI) were calculated using the delta method. Each model was accompanied by the Akaike's information criterion (AIC), a goodness-of-fit estimate recommended for exploratory analyses and comparison of complex models that penalizes for added model parameters (Aho et al., 2014).

Given that many recently-arrived respondents were missing register data from two years prior to each survey, we conducted sensitivity analyses excluding respondents with missing register data to explore the robustness of our findings. We additionally examined whether excluding panel respondents would influence our findings.

## 3 Results

The sample (n = 58,428; Table 1) was primarily comprised of Swedish-born respondents (n = 52,069), with fewer OECD (n = 4042) and non-OECD (n = 2317) migrant respondents. Approximately 55% of all respondents were women. Most migrants from OECD and non-OECD regions arrived in Sweden in early adulthood (25–34 years). OECD migrants were more likely than non-OECD migrants to have resided in Sweden for more than 15 years. Nearly one quarter of non-OECD migrants reported having psychological distress, followed by up to a fifth of OECD migrants and even fewer Swedish-born. A clear socioeconomic gradient of decreasing educational attainment and increasing income emerged across the nativity categories, from non-OECD to OECD migrants and finally to Swedish-born, with more negatively skewed income distributions among women of all nativities. Migrants experienced less social cohesion and increased discrimination relative to Swedish-born.

**Table 1:** Descriptive characteristics (n = 58,428; 2011 and 2015 cross-sectional, plus 2015 panel respondents).

		Men *n (%)*			Women *n (%)*		
		Swedish-born (n = 23,358)	OECD migrants (n = 1,720)	Non-OECD migrants (n = 1,096)	Swedish-born (n = 28,711)	OECD migrants (n = 2,322)	Non-OECD migrants (n = 1,221)
Survey year	2011	10,547 (45.2)	787 (45.8)	445 (40.6)	12,950 (45.1)	1,028 (44.3)	496 (40.6)
	2015	12,811 (54.9)	933 (54.2)	651 (58.4)	15,761 (54.9)	1,294 (55.7)	725 (59.4)
Age at survey	*Mean (SD)*	44.16 (14.06)	46.63 (12.59)	41.61 (13.50)	43.34 (14.00)	46.56 (12.72)	39.85 (12.25)
Age at migration	0–12 years		461 (26.8)	187 (17.1)		547 (23.6)	272 (22.3)
	13–17 years		117 (6.8)	95 (8.7)		175 (7.5)	81 (6.6)
	18–24 years		271 (15.8)	167 (15.2)		512 (22.1)	205 (16.8)
	25–34 years		451 (26.2)	408 (37.2)		630 (27.1)	385 (31.5)
	35–64 years		420 (24.4)	239 (21.8)		458 (19.7)	278 (22.8)
Duration of residence	≥15 years		1,073 (62.4)	557 (50.8)		1,513 (65.2)	590 (48.3)
**Mental health**							
Distress	GHQ-12 score >3	2,866 (12.3)	265 (15.4)	257 (23.5)	5,337 (18.6)	497 (21.4)	295 (24.2)
**Socioeconomic status**							
Educational level	Low	10,590 (45.3)	748 (43.5)	406 (37.0)	10,088 (35.1)	866 (37.3)	427 (35.0)
	Medium	8,768 (37.5)	535 (31.1)	325 (29.7)	11,221 (39.1)	708 (30.5)	380 (31.1)
	High	3,617 (15.5)	345 (20.1)	245 (22.4)	7,014 (24.4)	638 (27.5)	309 (25.3)
	*Unrecorded/missing*	383 (1.6)	92 (5.4)	120 (11.0)	388 (1.4)	110 (4.7)	105 (8.6)
Individual disposable income	Lowest Quintile	4,035 (17.3)	400 (23.3)	345 (31.5)	5,688 (19.8)	558 (24.0)	485 (39.7)
	Highest Quintile	7,333 (31.4)	368 (21.4)	117 (10.7)	3,657 (12.7)	244 (10.5)	60 (4.9)
	*Unrecorded/missing*	10 (0.0)	53 (3.1)	77 (7.0)	12 (0.0)	71 (3.0)	57 (4.6)
Household disposable income	Lowest Quintile	4,177 (17.9)	483 (28.1)	367 (33.5)	5,428 (18.9)	629 (27.1)	393 (32.2)
	Highest Quintile	5,040 (21.6)	248 (14.4)	107 (9.8)	5,900 (20.6)	307 (13.2)	110 (9.0)
	*Unrecorded/missing*	26 (0.1)	55 (3.2)	80 (7.3)	24 (0.1)	71 (3.1)	59 (4.8)
Civil status	Single	13,204 (56.5)	816 (47.4)	503 (45.9)	15,373 (53.5)	1,062 (45.7)	541 (44.3)
	Married or cohabiting	10,154 (43.5)	904 (52.6)	593 (54.1)	13,338 (46.5)	1,260 (54.3)	680 (55.7)
Citizenship	Swedish	23,280 (99.7)	1,022 (59.4)	768 (70.0)	28,614 (99.7)	1,458 (62.8)	857 (70.2)
**Social cohesion**							
Received practical support		22,457 (96.1)	1,559 (90.6)	881 (80.4)	27,983 (97.5)	2,139 (92.1)	1,060 (86.8)
Received emotional support		20,489 (87.7)	1,420 (82.6)	764 (69.7)	26,642 (92.8)	2,017 (86.9)	986 (90.8)
Generally trusts people		17,993 (77.0)	1,078 (62.7)	568 (51.8)	22,525 (78.5)	1,436 (61.8)	660 (54.1)
Participated in social activities		22,009 (94.2)	1,505 (87.5)	879 (80.2)	27,547 (96.0)	2,058 (88.6)	1,007 (82.5)
**Discrimination**							
Perceived discrimination		232 (1.0)	103 (6.0)	170 (15.5)	224 (0.8)	139 (6.0)	127 (10.4)
Feared going out alone		1,503 (6.4)	185 (10.8)	210 (19.2)	8,469 (29.5)	771 (33.2)	487 (40.0)
Exposed to threats		712 (3.1)	71 (4.1)	69 (6.3)	1290 (4.5)	129 (5.6)	74 (6.1)
Exposed to physical violence		712 (3.1)	62 (3.6)	57 (5.2)	770 (2.7)	59 (2.5)	38 (3.1)

**Fig. 1:**
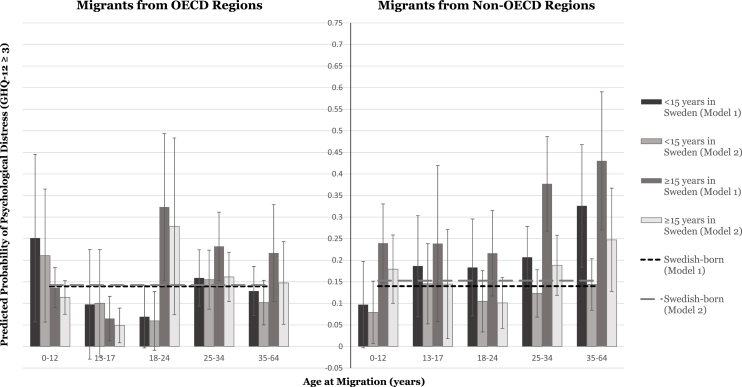
Predicted probabilities of psychological distress in migrant men by age at migration and duration of residence in Sweden, relative to Swedish-born men.

**Fig. 2:**
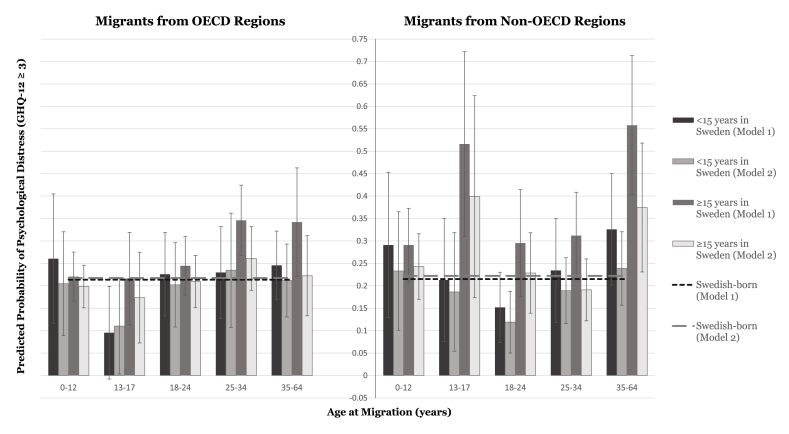
Predicted probabilities of psychological distress in migrant women by age at migration and duration of residence in Sweden, relative to Swedish-born women.

The results of the logistic regression analysis are illustrated as predicted probabilities in Fig. 1, Fig. 2 and reported as AMEs in Table 2, Table 3.

**Table 2:** Average marginal effects (AME) of nativity, age at migration, and duration of residence on psychological distress among men.

	Table 2i. Migrants from OECD regions (n = 1,720) (ref. Swedish-born: n = 23,358) AME (95% CI)		Table 2ii. Migrants from non-OECD regions (n = 1,096) (ref. Swedish-born: n = 23,358) AME (95% CI)	
**All migrants**	***Model 1a***	***Model 1b***	***Model 1a***	***Model 1b***
*All migrants*	0.030 (−0.003–0.063)	−0.001 (−0.041–0.039)	**0.115 (0.074**–0.156)***	−0.001 (−0.032–0.029)
**Age at migration**	***Model 2a***	***Model 2b***	***Model 2a***	***Model 2b***
*Age 0–12 years*	0.015 (−0.036–0.066)	−0.012 (−0.054–0.030)	0.059 (−0.020–0.138)	−0.002 (−0.065–0.061)
*Age 13–17 years*	−0.061 (-0.122--0.001)*	−0.074 (-0.127--0.021)**	0.066 (−0.035–0.167)	0.003 (−0.076–0.083)
*Age 18–24 years*	0.087 (−0.038–0.211)	0.055 (−0.093–0.203)	0.062 (−0.013–0.138)	−0.044 (−0.093–0.004)
*Age 25–34 years*	0.049 (−0.003–0.100)	0.023 (−0.029–0.075)	**0.136 (0.069**–0.203)***	0.008 (−0.041–0.057)
*Age 35–64 years*	0.012 (−0.040–0.065)	−0.020 (−0.074–0.033)	**0.203 (0.087**–0.319)**	0.023 (−0.035–0.081)
**Duration of residence**	***Model 3a***	***Model 3b***	***Model 3a***	***Model 3b***
*<15 years residence*	0.002 (−0.038–0.042)	−0.019 (−0.066–0.027)	**0.082 (0.025**–0.139)**	−0.031 (−0.069–0.008)
*≥15 years residence*	**0.052 (0.004**–0.100)*	0.005 (−0.042–0.052)	**0.155 (0.099**–0.211)***	0.017 (−0.023–0.057)
**Age at migration x** **Duration of residence**	***Model 4a***	***Model 4b***	***Model 4a***	***Model 4b***
<15 years residence				
*Age 0–12 years*	0.112 (−0.082–0.306)	0.068 (−0.086–0.223)	−0.043 (−0.143–0.058)	**−0.074 (-0.147**–0.000)*
*Age 13–17 years*	−0.042 (−0.169–0.085)	−0.042 (−0.167–0.083)	0.047 (−0.070–0.164)	−0.007 (−0.101–0.087)
*Age 18–24 years*	−0.070 (−0.143–0.002)	−0.083 (-0.152--0.014)*	0.043 (−0.069–0.156)	−0.048 (−0.120–0.025)
*Age 25–34 years*	0.019 (−0.046–0.085)	0.013 (−0.057–0.082)	0.067 (−0.005–0.139)	−0.029 (−0.087–0.028)
*Age 35–64 years*	−0.011 (−0.068–0.047)	−0.041 (−0.094–0.013)	**0.186 (0.044**–0.328)*	−0.009 (−0.070–0.052)
≥15 years residence				
*Age 0–12 years*	−0.003 (−0.050–0.044)	−0.029 (−0.068–0.011)	**0.100 (0.008**–0.191)*	0.027 (−0.053–0.106)
*Age 13–17 years*	−0.075 (-0.127--0.023)**	−0.094 (-0.135--0.052)***	0.099 (−0.082–0.280)	−0.008 (−0.135–0.119)
*Age 18–24 years*	**0.184 (0.014**–0.354)*	0.136 (−0.070–0.342)	0.076 (−0.023–0.176)	−0.052 (−0.111–0.008)
*Age 25–34 years*	**0.093 (0.013**–0.172)*	0.019 (−0.039–0.077)	**0.237 (0.128**–0.347)***	0.036 (−0.034–0.106)
*Age 35–64 years*	0.077 (−0.035–0.190)	0.005 (−0.091–0.101)	**0.291 (0.131**–0.451)***	0.095 (−0.026–0.215)
**AIC**	589582.6	550721.5	616329.1	572966.3

Models 1-4a: Controlling for age at the time of the survey, panel membership.

Models 1–4b: Controlling for age at the time of the survey, panel membership, socioeconomic status (educational level, individual and household income, civil status, Swedish citizenship), social cohesion (availability of practical support, availability of emotional support, general trust in people, social participation), discrimination (perceived discrimination, fear of going out alone, exposure to threats, exposure to physical violence).

Akaike's Information Criterion (AIC) apply to Models 4a and 4b.

*p < 0.05, **p < 0.01, ***p < 0.001.

**Table 3:** Average marginal effects (AME) of nativity, age at migration, and duration of residence on psychological distress among women.

	Table 3i. Migrants from OECD regions (n = 2,322) (ref. Swedish-born: n = 28,711) AME (95% CI)		Table 3ii. Migrants from non-OECD regions (n = 1,221) (ref. Swedish-born: n = 28,711) AME (95% CI)	
**All migrants**	***Model 1a***	***Model 1b***	***Model 1a***	***Model 1b***
*All migrants*	**0.031 (0.000**–0.062)*	−0.005 (−0.040–0.030)	**0.074 (0.032**–0.115)***	0.008 (−0.028–0.043)
**Age at migration**	***Model 2a***	***Model 2b***	***Model 2a***	***Model 2b***
*Age 0–12 years*	0.013 (−0.038–0.065)	−0.017 (−0.062–0.028)	**0.078 (0.004**–0.153)*	0.021 (−0.045–0.086)
*Age 13–17 years*	−0.058 (−0.141–0.025)	−0.070 (−0.147–0.006)	0.127 (−0.006–0.260)	0.063 (−0.069–0.195)
*Age 18–24 years*	0.024 (−0.030–0.077)	−0.008 (−0.060–0.044)	0.001 (−0.069–0.072)	−0.046 (−0.107–0.015)
*Age 25–34 years*	**0.067 (0.001**–0.133)*	0.033 (−0.042–0.108)	0.047 (−0.034–0.128)	−0.023 (−0.076–0.030)
*Age 35–64 years*	0.060 (−0.006–0.126)	0.001 (−0.063–0.065)	**0.156 (0.053**–0.258)**	0.056 (−0.017–0.130)
**Duration of residence**	***Model 3a***	***Model 3b***	***Model 3a***	***Model 3b***
*<15 years residence*	0.006 (−0.047–0.060)	−0.012 (−0.084–0.061)	0.034 (−0.028–0.096)	−0.022 (−0.072–0.027)
*≥15 years residence*	**0.050 (0.015**–0.086)**	−0.002 (−0.035–0.030)	**0.120 (0.066**–0.174)***	0.027 (−0.019–0.072)
**Age at migration x** **Duration of residence**	***Model 4a***	***Model 4b***	***Model 4a***	***Model 4b***
<15 years residence				
*Age 0–12 years*	0.047 (−0.098–0.191)	−0.013 (−0.129–0.104)	0.076 (−0.086–0.238)	0.011 (−0.122–0.144)
*Age 13–17 years*	−0.118 (-0.222--0.015)*	−0.107 (−0.215–0.000)	−0.002 (−0.140–0.135)	−0.036 (−0.169–0.097)
*Age 18–24 years*	0.012 (−0.081–0.106)	−0.015 (−0.110–0.080)	−0.063 (−0.142–0.016)	−0.103 (-0.173--0.033)**
*Age 25–34 years*	0.016 (−0.087–0.119)	0.017 (−0.112–0.146)	0.019 (−0.097–0.136)	−0.033 (−0.107–0.041)
*Age 35–64 years*	0.032 (−0.045–0.109)	−0.005 (−0.088–0.077)	0.111 (−0.014–0.235)	0.017 (−0.066–0.100)
≥15 years residence				
*Age 0–12 years*	0.007 (−0.048–0.062)	−0.019 (−0.067–0.029)	0.076 (−0.006–0.158)	0.021 (−0.053–0.095)
*Age 13–17 years*	0.003 (−0.100–0.106)	−0.044 (−0.145–0.058)	**0.301 (0.095**–0.507)**	0.177 (−0.048–0.402)
*Age 18–24 years*	0.031 (−0.035–0.097)	−0.008 (−0.067–0.051)	0.080 (−0.039–0.200)	0.006 (−0.084–0.097)
*Age 25–34 years*	**0.132 (0.054**–0.211)**	0.044 (−0.028–0.116)	0.097 (0.000–0.194)	−0.031 (−0.101–0.038)
*Age 35–64 years*	**0.128 (0.007**–0.250)*	0.005 (−0.085–0.095)	**0.343 (0.187**–0.499)***	**0.152 (0.009**–0.296)*
**AIC**	717813.2	679277.3	715763.8	673647.3

Models 1-4a: Controlling for age at the time of the survey, panel membership.

Models 1–4b: Controlling for age at the time of the survey, panel membership, socioeconomic status (educational level, individual and household income, civil status, Swedish citizenship), social cohesion (availability of practical support, availability of emotional support, general trust in people, social participation), discrimination (perceived discrimination, fear of going out alone, exposure to threats, exposure to physical violence).

Akaike's Information Criterion (AIC) apply to Models 4a and 4b.

*p < 0.05, **p < 0.01, ***p < 0.001.

We found a positive marginal effect of migrant status on the probability of psychological distress among men from OECD regions (AME 0.030 [95% CI -0.003–0.063]; Table 2i, **Model 1a**). The AME increased significantly with longer residence in Sweden (**Model 3a**). In jointly investigating age-at-migration and duration-of-residence categories, migrants arriving as children appeared to become increasingly protected against distress with time (e.g., *arrived aged 13–17:* −0.042 [-0.169–0.085] for *less than* and −0.075 [-0.127–0.023] for *more than 15 years' residence*), whereas migrants arriving after age 18 had increased AMEs with longer residence (e.g., *arrived aged 25–34:* 0.019 [-0.046–0.085] for *less than* and 0.093 [0.013–0.172] for *more than 15 years' residence*; **Model 4a**). Among men arriving from non-OECD regions, we found a significantly positive AME of overall migrant status on the probability of distress (0.115 [0.074–0.156]; Table 2ii, **Model 1a**). AMEs were greatest for those arriving after age 25 (e.g., *arrived aged 25–34:* 0.136 [0.069–0.203]; **Model 2a**) and increased with longer residence in Sweden (e.g., *arrived aged 35–64:* 0.186 [0.044–0.328] for *less than* and 0.291 [0.131–0.451] for *more than 15 years'* *residence*; **Model 4a**).

Women from OECD regions also had a positive AME of migrant status on the probability of psychological distress, both overall (AME 0.031 [95% CI 0.000–0.062]; Table 3i, **Model 1a**) and specifically among those arriving after age 25 (e.g., *arrived aged 25–34*: 0.067 [0.001–0.133]; **Model 2a**). As with their male counterparts, joint analyses revealed that women arriving as children from OECD regions were increasingly protected against distress with longer residence, with worse distress observed among all other age-at-migration groups (**Model 4a**). We found a greater marginal effect of migrant status on psychological distress among women from non-OECD regions (0.074 [0.032–0.115]; Table 3ii, **Model 1a**). For those who migrated less than 15 years prior, only women arriving as adolescents (*aged 13–17 years*: −0.002 [-0.140–0.135]) and young adults (*aged 18–24 years*: −0.063 [-0.142–0.016]) exhibited positive marginal effects on psychological distress. AMEs also increased with time in Sweden, particularly for those that migrated in adolescence (*aged 13–17 years*: 0.301 [0.095–0.507]) and later adulthood (*aged 35–64 years*: 0.343 [0.187–0.499]; **Model 4a**).

For all comparative groups, the reported AICs revealed that the fully-adjusted **Model 4b** was more parsimonious (i.e., had a lower value) than **Model 4a**, suggesting that the included social covariates improved the model fit (Table 2, Table 3). In many cases, these covariates explained the inequalities in psychological distress to the point of rendering the AMEs zero or even negative, with particularly substantial changes concentrated among non-OECD respondents and migrants with longer residence. Of these covariates, measures of social cohesion, followed by those of discrimination and SES, improved the goodness-of-fit to the greatest degree. More specifically, the largest improvement in the models' goodness-of-fit was attributable to the general trust measure, except for among OECD-origin men, whose greatest contribution came from social support (see Table S2 for respective AICs).

In our sensitivity analyses, excluding respondents with missing register data only slightly decreased the AME of recent migrant arrivals (e.g., from AME 0.186 [95% CI 0.044–0.328] to 0.143 [0.043–0.242] for non-OECD migrant men who arrived aged 35–64 years with less than 15 years' residence; Table S3). Excluding all panel respondents (i.e., those who participated in both 2011 and 2015 surveys) did not affect our findings (Table S4).

## 4 Discussion

This study examined differences in psychological distress by migrants' age at migration and duration of residence in Sweden, and whether such differences could be explained by various social determinants of health. We found that relative to native-born, probabilities of distress increased both with age at migration and duration of residence in Sweden. The greatest probabilities appeared in men and women originating from non-OECD regions. Native-migrant differences in social cohesion, and to a lesser degree those in SES and discrimination, appeared to explain a portion of these inequalities. These findings allude to smaller relative disadvantages in psychological health among younger arrivals, with increased exposure to social inequalities over time contributing to aggravated health across all ages at migration.

Although the *healthy migrant paradox* posits a protective effect of migrant status on physical health, evidence for mental health has been mixed (Abraido-Lanza et al., 1999; Kennedy et al., 2015). For instance, one European study identified poorer mental health among first-generation migrants, particularly those from outside Europe, relative to native-born (Levecque and Van Rossem, 2015). Similarly, we identified an overall disadvantage in mental health across most migrant groups, with migrants from non-OECD regions experiencing greater levels of psychological distress than their OECD-origin peers. This finding is even more profound considering the greater rates of poor mental health among the Swedish general population relative to other EU member states (European Commission, 2014).

Researchers hypothesize that these inequalities are a result of exposure to social and structural obstacles to integration, as well as unique threats to security through discrimination, all of which are particularly prevalent among persons from outside OECD-dominant regions (Levecque and Van Rossem, 2015; Solé-Auró and Crimmins, 2008). Revisiting our initial life course framework, it seems that exposure to such disadvantage during more sensitive life periods and over time would result in a chain or accumulation of risk for migrants regardless of background. This coincides with our finding of increased distress with age at migration (i.e., the initial sensitive period) and duration of residence (i.e., the period of accumulating risk).

Both older age at migration (Lanari and Bussini, 2012; Lanari et al., 2018) and increasing duration of residence (Lanari and Bussini, 2012) have been suggested to impact migrant psychological health. Yet to our knowledge, only one prior study has examined the joint effects of these two measures in Europe (Mood et al., 2016). The life course-oriented study revealed that adolescent migrants across Sweden, England, Germany, and the Netherlands experienced better psychological health than native-born, independent of their age at migration and region of origin, a finding similar to that of our younger male arrivals from OECD regions. Yet this multinational study did not consider adult arrivals, who according to our results experienced the greatest burden of psychological distress. We also found changes in mental health over a longer time span, suggesting that the advantages observed within the follow-up of the previous study may only be temporary.

Native-migrant health inequalities have generally been shown to decrease or *converge* over time as a consequence of sociocultural assimilation (Antecol and Bedard, 2006; Nader and Oliver, 2018; Yang, 2019). However, the bulk of convergence findings either relate to physical health outcomes in Sweden (Juárez et al., 2018) or originate from North American research (Alegría et al., 2007; Antecol and Bedard, 2006; Cook et al., 2009; Li and Wen, 2015; Yang, 2019), lending a contextual specificity to their conclusions. Due to its generous migration policy, Sweden may be subject to unique selection mechanisms, potentially allowing vulnerable populations to migrate later in adulthood where other contexts may have more exclusively selected for young, healthy labor migrants. Indeed, while we found that the youngest men and women from OECD regions converged to lower native levels of psychological distress with longer residence, most other studied cohorts appeared to diverge with increasing probabilities of distress, as has been previously observed in Sweden (Johnson et al., 2017). These findings support the *cumulative disadvantage theory*, describing the collective divergence of population characteristics such as health due to various social or structural forces (Dannefer, 2003), or, in our case, the cumulative effects of social determinants of health arising throughout integration (Ager and Strang, 2008).

Of these social determinants, SES has been previously shown to drive Swedish native-migrant mortality differences (Rostila and Fritzell, 2014). In our study, SES also appeared to partially modify the association between the timing of migration and psychological distress. Through the life course lens, we can postulate that the integration opportunities of early school enrolment and subsequent educational and occupational trajectories may decrease socioeconomic differences between young migrants and natives, contributing to a diminished sense of relative deprivation compared to those migrating later in adulthood (Åslund et al., 2009). Despite their high levels of education abroad, older migrant arrivals are more likely to be restricted entry into the Swedish labor market, leading to so-called educational mismatch or over-qualification for available jobs (Dahlstedt, 2011; Dunlavy et al., 2016) and an accumulation of socioeconomic disadvantage over time contributing to psychological distress.

We also found that health differences in the studied cohorts were strongly explained by measures of social cohesion, as seen previously (Johnson et al., 2017; Yang, 2019). Without sources of trust and support, young migrants with an already precarious sense of identity can develop symptoms of acculturative stress (Berry et al., 1987), potentially explaining how the early life advantages observed among non-OECD migrants diminish over time in Sweden. In older age-at-migration groups, social connections additionally serve as informal job networks (Edin et al., 2003), protecting against socioeconomic stressors and ultimately psychological distress. Thus, our finding of lower sources of support and trust among migrants emphasizes a crucial limitation in their integration trajectories.

Finally, we measured experiences of discrimination to understand how insecurity could contribute to native-migrant inequalities. Indeed, the measures partially attenuated our observed differences in psychological distress, as has been previously substantiated in Sweden (Wamala et al., 2007) and internationally (Pascoe and Smart Richman, 2009). This suggests that cumulative threats to security, socioeconomic stability, and social cohesion across the life course can become embodied and manifest as psychological distress, potentially leading to even more severe mental health phenomena in the long term.

### 4.1 Strengths and limitations

To our knowledge, ours is the first study to examine migrant psychological distress by both age at migration and duration of residence in Sweden. Although the study examined a specific Swedish region, the concentration of migrants is similar to that of the rest of the country, making our findings nationally generalizable.

Register-linked survey data enabled us to capture more moderate and foundational psychological symptoms than large-scale register data alone. Our decision to analyze the GHQ-12 was driven by the survey's data availability and prior applications in migrant research, although the measure remains to be validated among migrant populations, with a possibility of response bias stemming from cultural differences in attitudes towards psychological health. Indeed, respondents were less likely to fully complete the GHQ-12 if they had originated from non-OECD regions, arrived at an older age, and resided in Sweden for less time, with lower SES. All surveys were administered in Swedish without translation services (excepting web surveys, which were available in English and comprised 15% of responses), likely contributing to the high non-response rate of non-OECD migrants. Thus, we may have underestimated the health burden within this population, although we attempted to address any bias through inverse probability weighting. Rather than apply multiple imputation to address missing register data (Sterne et al., 2009), we included the missing values in our main analysis based on the assumption that they reflected systematic, not random, differences in coverage (i.e., for recent migrants from non-OECD contexts with potentially delayed registration). Sensitivity analyses confirmed that this choice did not affect our results.

The cross-sectional design restricted our ability to establish temporality and, in turn, negate the possibility of reverse causality between the timing of migration and psychological distress (i.e., whether psychological distress influenced the decision to migrate in the first place) and establish the exact sequence of exposures to identify relevant life course models. However, the panel data alone would have provided insufficient statistical power to maintain discrete age-at-migration and duration-of-residence categories and capture their potential effect-measure modification on psychological distress (Rothman, 2012).

Due to similar data limitations, we could neither distinguish second-generation migrants among Swedish-born nor identify reasons for migration. To address the latter, we created broad groupings of migrants based on OECD-region membership to proxy reasons for migration while maintaining statistical power. Supporting data on pre-migration experiences was missing, but could have helped to identify contributing factors to mental health and possible selection effects in the country of origin (Akresh and Frank, 2008). Information on migrants' exact date of arrival in Sweden was also unavailable, possibly skewing the interpretation of findings for migrants arriving as asylum seekers as they may have waited for over a year to be processed as a resident (Swedish Migration Agency, 2016).

Finally, we were only briefly able to report the role of various social determinants of health in dictating these associations. Due to the unexplored nature of these integration factors, we chose not to conduct an explicit mediation analysis. Instead, the purpose of this study was to explore, rather than quantify, the potential mediating role of each factor in the link between the timing of migration and psychological health.

## 5 Conclusion

In light of this study's findings, the Swedish state should aim to increase socioeconomic and cultural opportunities for integration so as to promote the long-term wellbeing of its migrants. This is especially true for migrants arriving later in life, who appear to lack the opportunities for integration found amongst their younger peers. Through these efforts, policymakers and other relevant actors can help to reinforce a sense of societal solidarity to facilitate native-migrant social cohesion. Adopting a preventative stance to address health inequalities early in integration can ultimately help to prevent the accumulation of social disadvantage and the gradual widening of health inequalities over time.

## CRediT authorship contribution statement

**Helena Honkaniemi:** Conceptualization, Data curation, Formal analysis, Methodology, Visualization, Writing - original draft, Writing - review & editing. **Sol Pía Juárez:** Conceptualization, Methodology, Project administration, Supervision, Writing - review & editing. **Srinivasa Vittal Katikireddi:** Methodology, Supervision, Writing - review & editing. **Mikael Rostila:** Conceptualization, Funding acquisition, Methodology, Project administration, Supervision, Writing - review & editing.
