# Supplementary material for: Psychological distress by age at migration and duration of residence in Sweden
Source: Soc Sci Med. 2020 Apr;250:112869. doi: 10.1016/j.socscimed.2020.112869 (PMC8325349; doi:10.1016/j.socscimed.2020.112869)
Supplement: Multimedia component 1 [file mmc1.docx]

**SUPPLEMENTARY FILES**

**2011**

**HET Supplementary Sample (VG): National Form (n=19,048)**

**2011**

**HET Supplementary Sample (VG): Regional Test Form (n=76,587)**

**2015**

**HET National Sample**

**(n=20,000)**

**2015**

**HET Supplementary Sample (VG)**

**(n=91,347)**

National respondents residing in VG

(n=3,297)

Supplementary VG respondents

(53.7%; n=49,051)

Total respondents in VG

(55.5%; n=52,348)

National Form respondents in VG (53.4%; n=10,190)

Regional Test Form respondents in VG (54.5%; n=41,740)

Total respondents in VG

(54.3%; n=51,930)

2011, 2015

Panel respondents (n=11,817)

2011

Cross-sectional sample

(n=40,113)

2015

Cross-sectional sample

(n=40,531)

Total non-respondents

(49.9%; n=103,273)

Missing information

Region of origin (n=2)

Age at arrival (n=299)

GHQ-12 (n=2,609)

Survey data (n=2,039)

Total sample

(All ages;

n=87,512)

National respondents

(48.8%; n=9,728)

Non-working age (65+ years; n=29,084)

Total sample

(Working age; n=58,428)

## **Figure S1. Participant selection**

***Table S1. “Health on Equal Terms” Survey Questions***

| **Domain** | **Variable** | **Swedish Original** | **English Translation** |
| --- | --- | --- | --- |
| **Social cohesion** | ***Instrumental (practical) support*** | Kan du få hjälp av någon/några personer om du har praktiska problem eller är sjuk? | Can you get help from other people if you have practical problems or are ill? |
|  | ***Emotional (social) support*** | Har du någon du kan dela dina innersta känslor med och anförtro dig åt? | Do you have anyone you can share your innermost feelings with and confide in? |
|  | ***Generalized trust*** | Tycker du att man i allmänhet kan lita på de flesta människor? | Do you believe in general that one can trust most people? |
|  | ***Social participation*** | Har du deltagit i någon av följande aktiviteter under de senaste 12 månaderna?  *Studiecirkel/kurs på din arbetsplats*  *Studiecirkel/kurs på din fritid*  *Fackföreningsmöte*  *Annat föreningsmöte*  *Teater/bio*  *Konstutställning*  *Religiös sammankomst*  *Sporttillställning*  *Skrivit insändare i tidning/tidskrift/blogg*  *Demonstration av något slag*  *Offentlig tillställning t.ex. nattklubb, danstillställning eller liknande*  *Större släktsammankomst*  *Privat fest*  *Chattat eller gjort inlägg på sociala nätverkssajter på internet*  *Deltagit i sociala nätverksaktiviteter på internet, t.ex. spel, diskussionsforum*  *Inget av ovanstående* | Have you participated in any of the following activities during the past 12 months?  *Study group/course at workplace*  *Study group/course during free time*  *Trade union meeting*  *Other association meeting*  *Theatre/cinema*  *Art exhibition*  *Religious gathering*  *Sports event*  *Written a submission to a newspaper/magazine/blog*  *Demonstration of some kind*  *Public event e.g., night club, dance event or the like*  *Larger family gathering*  *Private party*  *Chatted or posted on social networking sites on the internet*  *Participated in online social networking, e.g., games, discussion boards*  *None of the above* |
| **Discrimination** | ***Perceived discrimination*** | Har du under de senaste 3 månaderna blivit behandlad/bemött på ett sätt så att du känt dig kränkt? Hade den kränkande behandlingen/bemötandet samband med något av följande?  *Etnisk tillhörighet*  *Religion*  *Hudfärg* | In the past 3 months, have you been treated/discriminated against in a way that offended you? Did the offending treatment/discrimination relate to any of the following?  *Ethnic affiliation*  *Religion*  *Skin colour* |
|  | ***Fear of going out alone*** | Händer det att du avstår från att gå ut ensam av rädsla för att bli överfallen, rånad eller på annat sätt ofredad? | Do you ever refrain from going out alone for fear of being attacked, robbed, or otherwise molested? |
|  | ***Exposure to threats*** | Har du under de senaste 12 månaderna blivit utsatt för hot eller hotelser om våld så att du blev rädd? | In the past 12 months, have you been subjected to threats of physical violence so that you became afraid? |
|  | ***Exposure to physical violence*** | Har du under de senaste 12 månaderna blivit utsatt för fysiskt våld? | Have you been subjected to physical violence in the past 12 months? |

***Table S2. Akaike’s Information Criteria (AIC) for semi-adjusted*** ***logit models***

| **Included Covariates** | **Migrant men from OECD regions** | **Migrant men from non-OECD regions** | **Migrant women from OECD regions** | **Migrant women from non-OECD regions** |
| --- | --- | --- | --- | --- |
| ***Age at survey, panel membership*** (see Tables 2, 3; Model 4a) | 589582.6 | 616329.1 | 717813.2 | 715763.8 |
| **+ All Indicators of Socioeconomic Status** | 578670.4 | 604625.5 | 709700.6 | 707207.2 |
| + Educational level | 588764.1 | 616163.2 | 716870.9 | 714209.4 |
| + Individual disposable income | 583916.5 | 609109.4 | 715179.9 | 711910.4 |
| + Household disposable income | 584169.8 | 609590.6 | 713761.2 | 711208 |
| + Civil status | 587689 | 614048.7 | 713962.6 | 712448.5 |
| + Swedish citizenship | 589422.2 | 616275.2 | 717473.4 | 715391.7 |
| **+ All Indicators of Social Cohesion** | **564946.7** | **590448.4** | **689236.9** | **684462.2** |
| + Practical support | 578413.8 | 605187.1 | 706870 | 705502.8 |
| + Social support | **577525** | 604952.5 | 704857.6 | 701096.3 |
| + General trust in people | 579130.8 | **603328.3** | **702065.8** | **697772.3** |
| + Social participation | 586161.1 | 613466.7 | 716466.2 | 714302 |
| **+ All Indicators of Discrimination** | 576992.9 | 599641.6 | 705963.1 | 703305.7 |
| + Perceived discrimination | 586496.7 | 611092.4 | 714860.3 | 711595.3 |
| + Fear of going out alone | 581602.5 | 607869.9 | 715288.3 | 712908.5 |
| + Exposure to threats | 585582.2 | 610214.4 | 710263.6 | 709051.5 |
| + Exposure to physical violence | 587220.9 | 611887.2 | 715953.7 | 713620.9 |
| ***Fully-adjusted*** (see Tables 2, 3; Model 4b) | 550721.5 | 572966.3 | 679277.3 | 673647.3 |

Largest AIC differences by covariate group (i.e., socioeconomic status, social cohesion, discrimination) and by individual covariates designated in bold. AIC = Akaike’s Information Criterion.

***Table S3. Sensitivity Analysis: Average marginal effects (AME) of nativity, age at migration, and duration of residence on psychological distress, excluding respondents with missing register data***

| **Men** | **Migrants from OECD regions** (n=1,604)  (ref. Swedish-born: n=22,971)  AME (95% CI) | | **Migrants from non-OECD regions** (n=956)  (ref. Swedish-born: n=22,971)  AME (95% CI) | |  |  |
| --- | --- | --- | --- | --- | --- | --- |
| **Age at migration x**  **Duration of residence** | ***Model 1a*** | ***Model 1b*** | ***Model 2a*** | ***Model 2b*** |  |  |
| **<15 years residence** |  | |  | |  |  |
| *Age 0-12 years* | 0.077 (-0.140-0.295) | 0.026 (-0.113-0.166) | -0.034 (-0.153-0.086) | -0.068 (-0.151-0.015) |  |  |
| *Age 13-17 years* | 0.001 (-0.180-0.181) | -0.021 (-0.176-0.134) | -0.025 (-0.152-0.101) | -0.065 (-0.151-0.020) |  |  |
| *Age 18-24 years* | -0.058 (-0.144-0.028) | -0.076 (-0.154-0.001) | 0.014 (-0.105-0.133) | -0.057 (-0.136-0.021) |  |  |
| *Age 25-34 years* | 0.007 (-0.060-0.075) | -0.003 (-0.072-0.066) | 0.046 (-0.028-0.119) | -0.033 (-0.094-0.027) |  |  |
| *Age 35-64 years* | 0.001 (-0.067-0.070) | -0.042 (-0.098-0.014) | **0.143 (0.043-0.242)**** | -0.006 (-0.071-0.058) |  |  |
| **≥15 years residence** |  | |  | |  |  |
| *Age 0-12 years* | -0.010 (-0.056-0.036) | -0.034 (-0.073-0.005) | **0.103 (0.011-0.196)*** | 0.033 (-0.048-0.114) |  |  |
| *Age 13-17 years* | **-0.075 (-0.128--0.023)**** | **-0.094 (-0.136--0.053)***** | 0.097 (-0.083-0.277) | -0.008 (-0.133-0.117) |  |  |
| *Age 18-24 years* | **0.185 (0.015-0.355)*** | 0.137 (-0.070-0.345) | 0.076 (-0.024-0.175) | -0.051 (-0.110-0.008) |  |  |
| *Age 25-34 years* | **0.094 (0.014-0.174)*** | 0.019 (-0.040-0.078) | **0.238 (0.128-0.348)***** | 0.037 (-0.033-0.107) |  |  |
| *Age 35-64 years* | 0.080 (-0.034-0.193) | 0.005 (-0.092-0.102) | **0.292 (0.132-0.452)***** | 0.094 (-0.026-0.214) |  |  |
| **AIC** | 571586.9 | 534135.5 | 590341.2 | 549671 |  |  |
| **Women** | **Migrants from OECD regions** (n=2,196)  (ref. Swedish-born: n=28,317)  AME (95% CI) | | **Migrants from non-OECD regions** (n=1,103)  (ref. Swedish-born: n=28,317)  AME (95% CI) | |  |  |
| **Age at migration x**  **Duration of residence** | ***Model 3a*** | ***Model 3b*** | ***Model 4a*** | ***Model 4b*** |  |  |
| **<15 years residence** |  |  |  |  |  |  |
| *Age 0-12 years* | 0.058 (-0.107-0.224) | -0.014 (-0.144-0.117) | 0.106 (-0.097-0.308) | 0.023 (-0.124-0.170) |  |  |
| *Age 13-17 years* | **-0.124 (-0.236--0.012)*** | -0.118 (-0.239-0.003) | -0.001 (-0.173-0.171) | -0.082 (-0.206-0.042) |  |  |
| *Age 18-24 years* | 0.016 (-0.087-0.119) | -0.025 (-0.123-0.072) | -0.051 (-0.138-0.035) | **-0.105 (-0.177--0.032)**** |  |  |
| *Age 25-34 years* | 0.010 (-0.107-0.126) | -0.001 (-0.137-0.136) | 0.055 (-0.080-0.190) | -0.044 (-0.119-0.030) |  |  |
| *Age 35-64 years* | 0.043 (-0.040-0.126) | -0.001 (-0.083-0.081) | **0.163 (0.025-0.300)*** | 0.020 (-0.065-0.106) |  |  |
| **≥15 years residence** |  |  |  |  |  |  |
| *Age 0-12 years* | 0.009 (-0.047-0.064) | -0.017 (-0.066-0.032) | 0.083 (-0.001-0.166) | 0.027 (-0.048-0.103) |  |  |
| *Age 13-17 years* | 0.003 (-0.100-0.105) | -0.044 (-0.145-0.056) | **0.300 (0.094-0.506)**** | 0.177 (-0.048-0.403) |  |  |
| *Age 18-24 years* | 0.031 (-0.035-0.096) | -0.008 (-0.067-0.051) | 0.080 (-0.039-0.200) | 0.006 (0.084-0.096) |  |  |
| *Age 25-34 years* | **0.132 (0.054-0.211)**** | 0.043 (-0.029-0.115) | **0.098 (0.001-0.195)*** | -0.032 (-0.101-0.037) |  |  |
| *Age 35-64 years* | **0.129 (0.008-0.250)*** | 0.005 (-0.085-0.094) | **0.345 (0.190-0.501)***** | **0.150 (0.006-0.293)*** |  |  |
| **AIC** | 697805.7 | 660615.7 | 694786.5 | 655431.8 |  |  |

Models 1a-4a: Controlling for age at the time of the survey, panel membership. Models 1b-4b: Controlling for age at the time of the survey, panel membership, socioeconomic status (educational level, individual and household income, civil status, Swedish citizenship), social cohesion (availability of practical support, availability of emotional support, general trust in people, social participation), discrimination (perceived discrimination, fear of going out alone, exposure to threats, exposure to physical violence). AIC = Akaike’s Information Criterion. AME = Average marginal effect.

*p<0.05, **p<0.01, ***p<0.001

***Table S4. Sensitivity Analysis: Average marginal effects (AME) of nativity, age at migration, and duration of residence on psychological distress, excluding panel respondents***

| **Men** | **Migrants from OECD regions** (n=1,515)  (ref. Swedish-born: n=20,709)  AME (95% CI) | | **Migrants from non-OECD regions** (n=972)  (ref. Swedish-born: n=20,709)  AME (95% CI) | |  |  |
| --- | --- | --- | --- | --- | --- | --- |
| **Age at migration x**  **Duration of residence** | ***Model 1a*** | ***Model 1b*** | ***Model 2a*** | ***Model 2b*** |  |  |
| **<15 years residence** |  | |  | |  |  |
| *Age 0-12 years* | 0.112 (-0.082-0.307) | 0.068 (-0.086-0.223) | -0.043 (-0.143-0.058) | **-0.073 (-0.147-0.000)*** |  |  |
| *Age 13-17 years* | -0.042 (-0.169-0.086) | -0.042 (-0.168-0.084) | 0.047 (-0.071-0.164) | -0.007 (-0.102-0.087) |  |  |
| *Age 18-24 years* | -0.071 (-0.144-0.002) | **-0.084 (-0.153--0.015)*** | 0.044 (-0.069-0.157) | -0.048 (-0.120-0.025) |  |  |
| *Age 25-34 years* | 0.019 (-0.047-0.085) | 0.013 (-0.057-0.083) | 0.067 (-0.005-0.139) | -0.029 (-0.087-0.028) |  |  |
| *Age 35-64 years* | -0.012 (-0.069-0.046) | -0.041 (-0.095-0.013) | **0.186 (0.043-0.329)*** | -0.009 (-0.071-0.052) |  |  |
| **≥15 years residence** |  | |  | |  |  |
| *Age 0-12 years* | -0.003 (-0.050-0.044) | -0.029 (-0.069-0.011) | **0.100 (0.008-0.192)*** | 0.027 (-0.053-0.107) |  |  |
| *Age 13-17 years* | **-0.076 (-0.128--0.024)**** | **-0.094 (-0.135--0.053)***** | 0.098 (-0.083-0.280) | -0.008 (-0.136-0.119) |  |  |
| *Age 18-24 years* | **0.185 (0.014-0.356)*** | 0.137 (-0.070-0.344) | 0.077 (-0.023-0.177) | -0.052 (-0.112-0.008) |  |  |
| *Age 25-34 years* | **0.093 (0.013-0.173)*** | 0.019 (-0.039-0.077) | **0.238 (0.127-0.348)***** | 0.036 (-0.035-0.107) |  |  |
| *Age 35-64 years* | 0.076 (-0.038-0.190) | 0.004 (-0.093-0.102) | **0.290 (0.130-0.451)***** | 0.094 (-0.026-0.215) |  |  |
| **AIC** | 586254.5 | 547612 | 613076 | 569914.7 |  |  |
| **Women** | **Migrants from OECD regions** (n=2,044)  (ref. Swedish-born: n=25,137)  AME (95% CI) | | **Migrants from non-OECD regions** (n=1,061)  (ref. Swedish-born: n=25,137)  AME (95% CI) | |  |  |
| **Age at migration x**  **Duration of residence** | ***Model 3a*** | ***Model 3b*** | ***Model 4a*** | ***Model 4b*** |  |  |
| **<15 years residence** |  |  |  |  |  |  |
| *Age 0-12 years* | 0.047 (-0.098-0.192) | -0.012 (-0.129-0.105) | 0.077 (-0.086-0.240) | 0.012 (-0.121-0.145) |  |  |
| *Age 13-17 years* | **-0.119 (-0.222--0.015)*** | -0.108 (-0.215-0.000) | -0.001 (-0.139-0.137) | -0.035 (-0.169-0.099) |  |  |
| *Age 18-24 years* | 0.013 (-0.081-0.107) | -0.014 (-0.110-0.082) | -0.063 (-0.142-0.016) | **-0.103 (-0.173--0.033)**** |  |  |
| *Age 25-34 years* | 0.017 (-0.086-0.120) | 0.018 (-0.111-0.148) | 0.019 (-0.097-0.136) | -0.033 (-0.107-0.042) |  |  |
| *Age 35-64 years* | 0.032 (-0.045-0.109) | -0.005 (-0.088-0.079) | 0.111 (-0.014-0.236) | 0.017 (-0.066-0.101) |  |  |
| **≥15 years residence** |  |  |  |  |  |  |
| *Age 0-12 years* | 0.006 (-0.050-0.062) | -0.020 (-0.068-0.029) | 0.076 (-0.007-0.159) | 0.021 (-0.053-0.095) |  |  |
| *Age 13-17 years* | 0.003 (-0.100-0.107) | -0.043 (-0.145-0.059) | **0.302 (0.095-0.510)**** | 0.179 (-0.048-0.405) |  |  |
| *Age 18-24 years* | 0.031 (-0.035-0.097) | -0.008 (-0.067-0.052) | 0.081 (-0.040-0.201) | 0.007 (-0.084-0.098) |  |  |
| *Age 25-34 years* | **0.133 (0.054-0.212)**** | 0.044 (-0.028-0.116) | 0.097 (-0.001-0.194) | -0.031 (-0.101-0.039) |  |  |
| *Age 35-64 years* | **0.130 (0.007-0.252)*** | 0.006 (-0.085-0.098) | **0.343 (0.187-0.498)***** | **0.152 (0.008-0.296)*** |  |  |
| **AIC** | 713404.2 | 675092.7 | 711429.4 | 669552.3 |  |  |

Models 1a-4a: Controlling for age at the time of the survey. Models 1b-4b: Controlling for age at the time of the survey, socioeconomic status (educational level, individual and household income, civil status, Swedish citizenship), social cohesion (availability of practical support, availability of emotional support, general trust in people, social participation), discrimination (perceived discrimination, fear of going out alone, exposure to threats, exposure to physical violence). AIC = Akaike’s Information Criterion. AME = Average marginal effect.

*p<0.05, **p<0.01, ***p<0.001
